# Radiotherapy: the key to immunotherapy ignition?

**DOI:** 10.18632/oncotarget.22070

**Published:** 2017-10-25

**Authors:** Elaine Luterstein, Narek Shaverdian, Percy Lee

**Affiliations:** Percy Lee: Department of Radiation Oncology, David Geffen School of Medicine at UCLA, Los Angeles, CA, USA

**Keywords:** radiotherapy, immunotherapy, check point inhibition, synergy, non-small cell lung cancer

In recent years, advances in immunotherapeutic approaches for advanced non-small cell lung cancer (NSCLC) treatment have shown promising results in both preclinical and clinical studies. Currently, it is believed that high programmed-death ligand (PD-L1) expression is a predictor of response to check-point inhibitors for NSCLC, but only approximately 20% of patients with NSCLC have high PD-L1 expression [1]. This emphasizes the necessity of finding novel approaches to boost the effectiveness of checkpoint inhibitors across all patient subgroups.

Immunotherapy drugs targeting the PD-1/PD-L1 checkpoint pathway disrupt inhibitory signaling allowing for increased anti-tumor immune responses. Based on preclinical studies, it has been postulated that radiation of a tumor releases tumor-associated antigens and stimulatory molecules, enhances antigen presentation and promotes antigen diversity, therefore potentially enhancing the generation of antitumor immunity [2]. Thus radiotherapy may therefore allow for increased clinical activity of checkpoint inhibitors by better priming the antitumor immune response.

The secondary analysis of patients with advanced NSCLC treated in the KEYNOTE-001 trial at UCLA investigated the impact of prior radiation therapy on the efficacy of pembrolizumab and substantiated prior preclinical findings of synergy between radiotherapy and immunotherapy. Out of 97 total patients, 42 received radiotherapy prior to immunotherapy, and both median progression-free survival and overall survival for this patient cohort were significantly longer than for patients without prior radiotherapy (progression-free and overall survival of 4.4 months vs 2.1 months, and 10.7 months vs 5.3 months, respectively) [3].

PD-L1 expression greater than 50% (high PD-L1) may be a predictor of response to lung cancer immunotherapies [1], but the patient cohorts from UCLA’s study provided evidence that prior radiotherapy superseded the importance of PD-L1 expression: patients who had received radiotherapy prior to being treated with pembrolizumab had lower baseline PD-L1 expression than patients without prior radiation (12% versus 22% high PD-L1) but longer median progression-free survival and overall survival [3]. These findings were replicated in another single-institution retrospective study of metastatic lung cancer patients treated with both thoracic radiotherapy and PD-1/PD-L1 inhibitors, which also found that thoracic radiotherapy prior to immunotherapy resulted in longer overall survival [4].

The results of the PACIFIC study parallel those of the UCLA secondary analysis of the KEYNOTE-001 trial. The PACIFIC study was a prospective study conducted using the PD-L1 inhibitor durvalumab as consolidative therapy for locally advanced (Stage IIIA, IIIB) NSCLC after conventional chemoradiotherapy, with patients randomized 2:1 to receive either consolidative immunotherapy or placebo [5]. The study, which examines the success of immunotherapy treatment, set 25% PD-L1 expression as the threshold and even then found that PD-L1 expression was not a predictive factor of response to anti-PD-L1 therapy after chemoradiotherapy.

Taken together, these findings might suggest PD-L1 status becomes less relevant after radiotherapy – which effectively eliminates response differences based on baseline PD-L1 expression – and provoke reevaluation of the role of PD-L1 in the context of radiotherapy in NSCLC. The upcoming phase I/II study evaluating the combination of stereotactic body radiotherapy and durvalumab in early-stage NSCLC patients at UCLA (NCT03148327) intends to further investigate this activity of radiotherapy in combination with immunotherapy and aims to expand the role of this treatment combination in early stage disease.

With the predictive value of PD-L1 expression under scrutiny, the matter of identifying a predictive biomarker for immunotherapy response remains. Immune function status and levels of key pro-cytotoxic cytokines including interferon gamma (IFN-γ) may be better biomarkers. In a Phase I/II study evaluating response rate to durvalumab in 228 NSCLC patients, baseline expression of IFN-γ correlated with increased response rate. Patients expressing IFN-γ showed a response rate of 33% to durvalumab, in contrast to IFN-γ negative patients, who exhibited a response rate of just 8% [6]. This promising result must be further validated with other anti-PD-1/PD-L1 therapies and further work is needed to identify panels of cytokines that can best serve as predictive biomarkers.

In this context, radiotherapy no longer exclusively serves as a local therapy constrained to locoregional control – instead, this potentially paradigm-changing approach allows radiotherapy to become an immuno-adjuvant therapy. As a boost to the immune anti-tumor response with system-wide effects, radiotherapy carries the potential to sensitize patients to immunotherapy treatments and convert traditionally non-responsive patents to treatment responders. As immunotherapy approaches continue to develop, these premises must be further corroborated with data. It is essential to understand the interplay between radiotherapy and immunotherapy (radiotherapy type, dose, target, timing, and immune anti-tumor response) by performing carefully designed clinical trials, given that these early findings highlight a novel role for radiotherapy.
